# Efficacy and safety of intermittent theta burst stimulation (iTBS) in treatment resistant depression

**DOI:** 10.1192/j.eurpsy.2022.263

**Published:** 2022-09-01

**Authors:** E. Kavakbasi, M. Tonkul, B. Baune

**Affiliations:** University Hospital Muenster, Department Of Psychiatry, Münster, Germany

**Keywords:** treatment resistant depression, iTBS, TMS, TRD

## Abstract

**Introduction:**

Repetitive transcranial magnetic stimulation (rTMS) is a non-invasive neuromodulatory treatment option, which is used in a variety of neurological and psychiatric diseases. It is approved for depression treatment and recommended in international guidelines. A significant reduction in treatment duration was achieved by using theta burst stimulation (TBS) protocols, which are practicable and non-inferior to conventional TMS.

**Objectives:**

To analyse the efficacy and safety of intermittent theta burst stimulation (iTBS) of left DLPFC in inpatients with treatment-resistant depression.

**Methods:**

We evaluated n=44 inpatients with treatment resistant major depressive disorder (n=37) and bipolar depression (n=7), who were treated with the 5 Hz intermittent TBS once daily for 3-6 weeks according to clinical decision. A total of 600 pulses and 200 bursts were applied in each treatment session. Clinical and response data were obtained by chart review.

**Results:**

Mean age at time of first stimulation was 54 years. 61,3 % of patients were female. On average, the current episode started 21 months before the first stimulation. In total, 924 treatment sessions were performed. On average, patients received 21 sessions. The mean MADRS Score pre-treatment was 27.2. Post-treatment, there was a clear reduction in depression severity (MADRS 18.3). No severe adverse events and no seizures occurred in this clinical observational analysis.

**Conclusions:**

Intermittent TBS is efficacious and safe in patients with chronic and refractory depression.

**Disclosure:**

No significant relationships.

